# Malignant Mesothelioma of the Pericardium: A Report of Two Different Presentations

**DOI:** 10.1155/2013/356901

**Published:** 2013-08-21

**Authors:** Pattarapong Makarawate, Narumol Chaosuwannakit, Jarin Chindaprasirt, Piti Ungarreevittaya, Surachat Chaiwiriyakul, Kosin Wirasorn, Chusak Kuptarnond, Kittisak Sawanyawisuth

**Affiliations:** ^1^Department of Medicine, Faculty of Medicine, Khon Kaen University, Khon Kaen 40002, Thailand; ^2^Department of Radiology, Faculty of Medicine, Khon Kaen University, Khon Kaen 40002, Thailand; ^3^Division of Oncology, Department of Medicine, Faculty of Medicine, Khon Kaen University, Khon Kaen 40002, Thailand; ^4^Department of Pathology, Faculty of Medicine, Khon Kaen University, Khon Kaen 40002, Thailand; ^5^Department of Surgery, Faculty of Medicine, Khon Kaen University, Khon Kaen 40002, Thailand; ^6^The Research and Training Center for Enhancing Quality of Life of Working-Age People, Khon Kaen University, Khon Kaen 40002, Thailand

## Abstract

Malignant mesothelioma of the pericardium is a rare and fatal condition that clinicians should be aware of due to its variability of clinical manifestation. The diagnosis may be delayed as a result of delayed treatment. Here, we report two cases of malignant pericardial mesothelioma with two different clinical aspects: cardiac tamponade and mimic tuberculous pericarditis. Both patients: may have indirect exposure to asbestos. Despite chemotherapy, both patients died at 2 weeks and 3 months after the diagnosis. Malignant mesothelioma of the pericardium is fatal, has a variety of presentation, and may not be related to asbestosis exposure.

## 1. Introduction

Malignant pericardial mesothelioma is extremely rare and has poor prognosis. It accounted for only 0.3% of mesothelioma death worldwide [1]. Unlike pleural mesothelioma, the relationship between asbestos and this lethal tumor is unclear [2]. Patients usually present with dyspnea on exertion caused by pericardial effusion or heart failure. The characters of pericardial effusion, however, may be different such as cardiac tamponade or constrictive pericarditis [3]. Here, we reported two patients diagnosed as pericardial malignant mesothelioma with two different presentations. 

## 2. Case Presentations

### 2.1. Case  1

**Figure 1:**
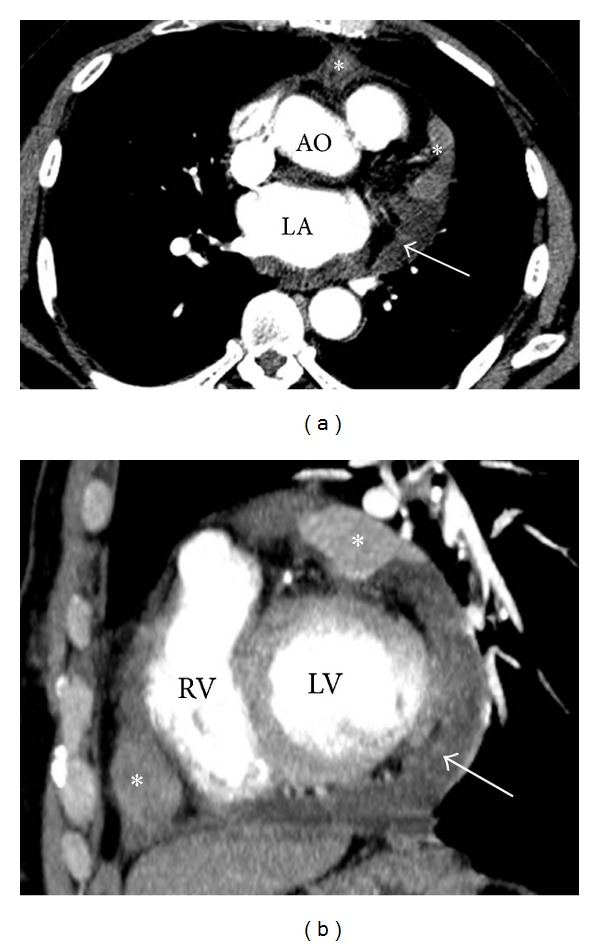
Axial (a) and sagittal (b) contrast-enhanced CT scan showed enhanced soft tissue nodules (asterisk) that have infiltrated the pericardium and moderate amount of pericardial effusion (arrow) (AO: aorta, LA: left atrium, RV: right ventricle, LV: left ventricle).

A 57-year-old man was transferred to our hospital on August 2011, with a two-month history of dyspnea on exertion and dizziness and three days of rapidly progressive dyspnea. On physical examination, his vital signs were stable but extra heart sound was heard. His past medical history was unremarkable apart from the fact that he worked as a road construction worker in Lampang province for 15 years. Electrocardiography showed generalized low voltage without ST segment change. Echocardiography was performed and showed a massive pericardial effusion, bright pericardium, and sign of early tamponade. CT scan revealed enhanced soft tissue nodules that have infiltrated the pericardium and moderate amount of pericardial effusion (Figure 1). 

**Figure 2:**
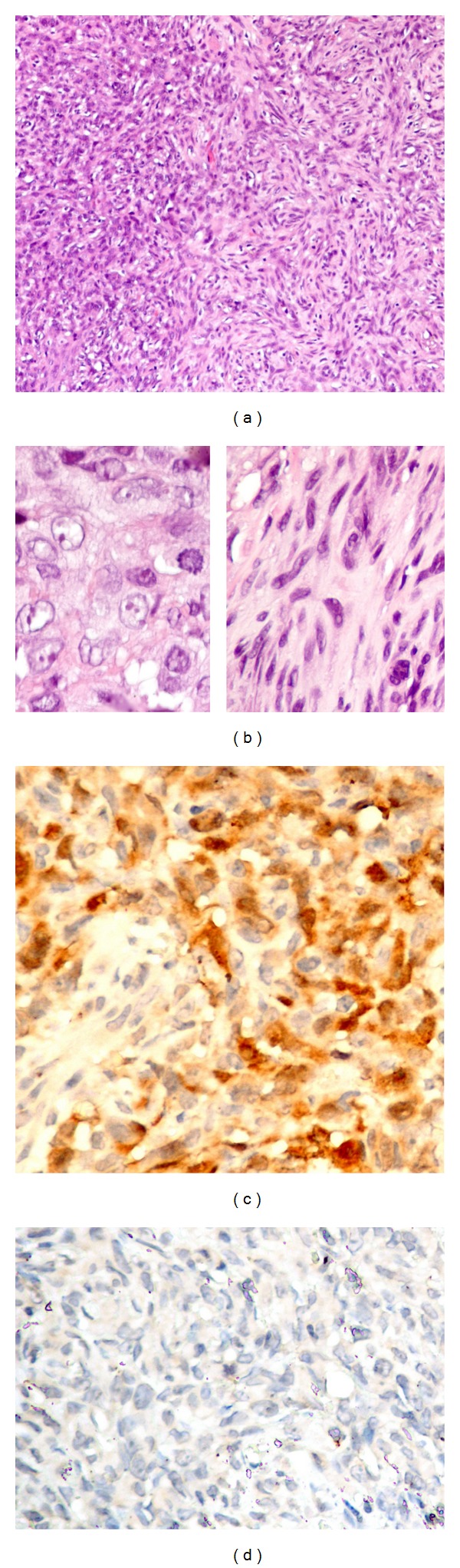
Malignant mesothelioma, biphasic type. A combination of epithelioid and sarcomatoid patterns (hematoxylin-eosin, original magnifications ×10 (a), ×100 (b)) and immunohistochemistry stains with calretinin (c) and TTF1 (d), original magnifications ×40. The neoplastic cells were arranged in sheets of epithelioid cells ((b), left) and interlacing fascicles of spindle cells ((b), right). Neoplastic cells expressed strong nuclear calretinin staining with heterogeneous cytoplasmic staining (c). Immunohistochemistry stain for TTF1 was negative (d).

The patient underwent pleuropericardial window procedure with pericardial and diaphragm nodule biopsy. The symptom of dyspnea was improved significantly after the surgery. Subsequent histopathological examination of the surgical specimen showed neoplastic cells containing both epithelioid and sarcomatoid patterns. The epithelioid cells are arranged in clusters. The sarcomatoid pattern consists of spindle cells arranged in fascicles and haphazard distribution. The malignant cells have oval nuclei, vesicular nucleus and distinct nuclei. Mitosis and necrosis are frequently seen (Figure 2). Immunohistochemistry shows positive mesothelial markers (calretinin and CK5/6) and negative pulmonary epithelial markers (CD15, TTF-1 and CEA). The other immunohistochemical markers were positive for pancytokeratin (AE1/AE3), EMA, CK7, CD99, and negative for CK20, vimentin, human muscle actin, myogenin, desmin, caldesmon, S-100, CD34, Bcl 2. Thus, a diagnosis of pericardial mesothelioma, biphasic type, was made. 

Since it was inoperable, the patient received palliative chemotherapy. After 4 cycles of pemetrexed (500 mg/m^2^) and cisplatin (75 mg/m^2^) given every 21 days, he refused further treatment. The dyspnea symptom was improved but he experienced asthenia and anorexia. The patient died at home three months later. 

### 2.2. Case  2

**Figure 3:**
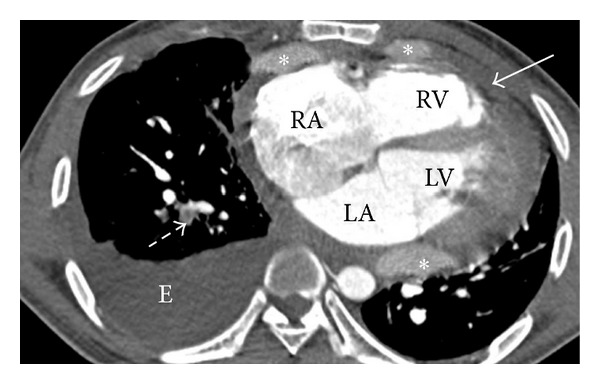
Axial contrast-enhanced CT scan showed enhanced soft tissue nodules (asterisk) that have infiltrated the pericardium, moderate amount of pericardial effusion (arrow), right pleural effusion (E), and partially occlusive clot in posterobasal segmental branch of right lower lobe pulmonary artery (dashed arrow) (RA: right atrium, LA: left atrium, RV: right ventricle, LV: left ventricle).

A 27-year-old man presented with chest pain and was treated as tuberculous pericarditis because he presented with constrictive pericarditis with lymphocytic pericardial effusion. After four months of antituberculous therapy, the effusion was increased and the patient was transferred to our hospital. His vital signs were stable with no sign of heart failure or cardiac tamponade. His past medical history was unremarkable. Echocardiography showed effusive constrictive pericarditis and thickening of pericardium with calcification. CT scan showed enhanced soft tissue nodules that have infiltrated the pericardium, moderate amount of pericardial effusion, and right pleural effusion (Figure 3). 

**Figure 4:**
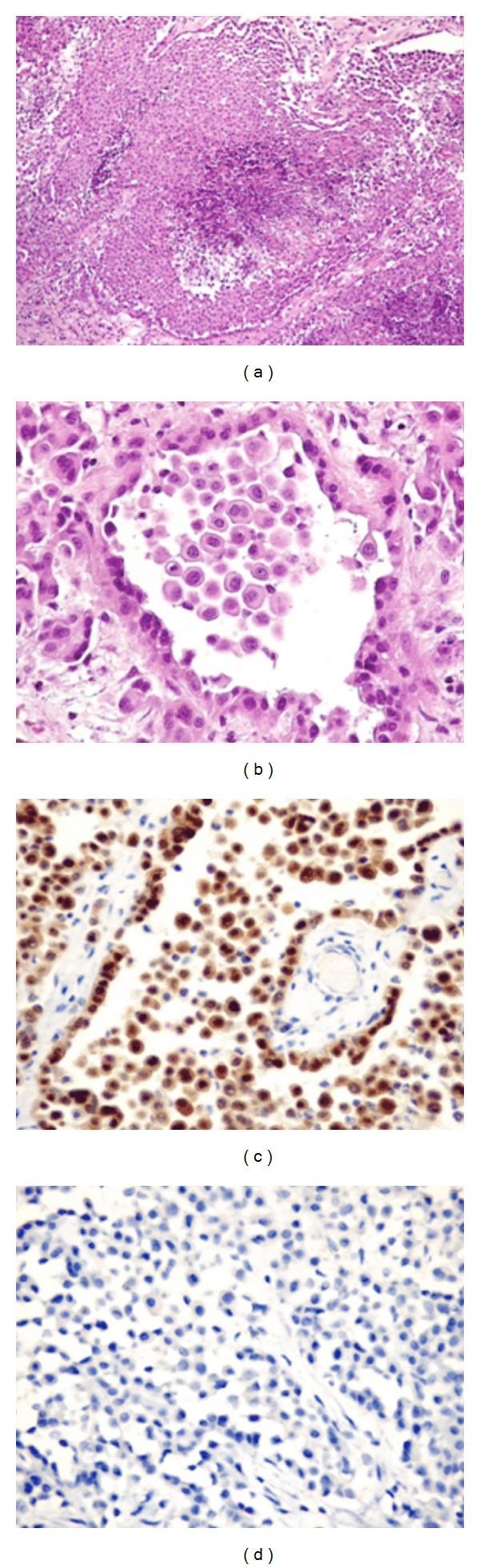
Photographs of malignant epithelioid mesothelioma (hematoxylin-eosin, original magnifications ×10 (a), ×40 (b)) and immunohistochemistry stains with calretinin (c) and CD15 (d), original magnifications ×40 ((c), (d)). The neoplastic cells were arranged in sheets with tumor necrosis at the center (a). Some areas showed large tubular and dyscohesive patterns (b). Neoplastic cells expressed strong nuclear calretinin staining with heterogeneous cytoplasmic staining (c). Immunohistochemistry stain for CD15 was negative (d).

The patient underwent pericardiectomy and mediastinal node biopsy. Intraoperative findings are 400 mL of turbid pericardial fluid and pericardial thickening (0.3 cm). Pathological examination of pericardium showed malignant epithelioid neoplastic cells arranged in sheets, large tubules, and discohesive patterns invading pericardium and lymphatic vessels. The mediastinal lymph node revealed metastatic neoplasm. Immunohistochemistry stains showed expression of calretinin, CK5/6, AE1/AE3, CK7, and vimentin by the neoplastic cells. Stains for carcinoembryonic antigen (CEA), CD15, and thyroid transcription factor-1 (TTF-1) are negative. The result was consistent with pericardial mesothelioma with mediastinal metastasis (Figure 4). 

Two weeks later, he developed hypoxemia caused by acute pulmonary embolism. During the course of chemotherapy (cisplatin and pemetrexed) the patient had a sudden cardiac arrest and died. 

## 3. Discussion

From January 1993 to September 2012, there are 60 patients diagnosed as mesothelioma. The rate of pericardial mesothelioma is 3.33%. Two reports from Japan showed that the pericardial mesothelioma associated with asbestos exposure was 0.7–0.8% of overall mesothelioma cases [1, 2]. The two reported cases had no history of direct exposure to asbestos. Both patients may have indirect asbestos exposure from road construction works and car wheel construction. Very few patients were reported as asbestos-related diseases, either asbestosis or asbestos-related lung cancer or mesothelioma [4]. This issue should be further addressed due to very high consumption of asbestos in Thailand. In the last decade, Thailand was the fifth importer of the world. The role of asbestos in pericardial mesothelioma, however, is unclear [2]. Asbestos may not be a cause of the second patient due to young age of onset. It may take 20–30 years of asbestos exposure to develop asbestosis or asbestos-related diseases. In addition, death from mesothelioma may be followed after 40 years of country asbestos consumption [1]. The incidence of asbestos-related diseases and mesothelioma may increase in Thailand in the near future. Physicians should be aware of these diseases.

Even though asbestos is the major cause of mesothelioma, asbestos body will not be found in the mesothelioma tumor mass. The diagnosis of mesothelioma may need a support from immunohistochemistry test. The positive calretinin and CK5/6 tests are suggestive of mesothelioma [5, 6]. In addition, computed tomography may be a helpful tool. Similar to these two patients and previous reports, CT scan showed enhanced pericardial nodules with pericardial effusion [7–10]. Pleural effusion may be or not be presented. Other diagnostic modalities are MRI or PET scan. As other malignancies, the definite diagnosis though is made by pathological findings.

There are some similar features of these both patients including long history of dyspnea symptom, enhanced nodular pericardial lesion, positive calretinin and CK5/6, and short survival after pemetrexed and cisplatin treatment (Table 1). The learning clinical point from our cases is that pericardial effusion from malignant mesothelioma may cause different cardiac manifestations cardiac tamponade in the first case: and mimic tuberculous pericarditis in the second case. Pathological diagnosis of tuberculous pericarditis may be required, but therapeutic trial may be done in Thailand where tuberculosis is common and pericardial biopsy may be difficult due to unavailability and invasiveness. The close clinical followup is needed. If pericardial effusion is not improved, further pericardial tissue is needed for definite diagnosis. A previous report also showed that malignant mesothelioma may mimic left atrial myxoma [11]. 

In terms of treatment, no standard treatment guideline for pericardial mesothelioma has been established yet. Surgical resection is the treatment of choice in localized disease. However, excision is not possible for those in locally advanced stage. Radiation has little effect on this tumor. Chemotherapy appeared once to be of little benefit for mesothelioma, but nowadays novel agents were developed and demonstrated to be effective [12]. Pemetrexed is a third generation antifolate drug. It is a promising agent often used with cisplatin or carboplatin to treat pleural mesothelioma since the combination improved progression-free and overall survival significantly [13]. Cisplatin combined with pemetrexed has demonstrated some activity against pleural mesothelioma and is also considered as first line in pericardial mesothelioma [13]. The outcomes of the combination in pericardial mesothelioma, however, vary in a few case reports [8, 14]. Both of our patients received cisplatin and pemetrexed but the results were not impressive.

## 4. Conclusion

Malignant mesothelioma of the pericardium is fatal, has a variety of presentation, and may not be related to asbestosis exposure. CT scan of enhanced soft tissue nodules infiltrating the pericardium may be a clue for diagnosis. 

## Figures and Tables

**Table 1:** Summary of two patients with malignant pericardial mesothelioma.

Clinical features	Case no. 1	Case no. 2
Age	57	26
Occupation	Road contractor	Car wheel construction
Duration of symptom	3 months	4 months
Provisional diagnosis	Cardiac tamponade	Tuberculous pericarditis
Pleural effusion	No	Yes
CT finding	Enhanced nodules in pericardium	Enhanced nodules in pericardium
Immunohistochemistry	Positive calretinin and CK5/6	Positive calretinin and CK5/6
Treatment	Pemetrexed and cisplatin	Pemetrexed and cisplatin
Survival	3 months	2 weeks
